# Estimating Post-Feeding Developmental Time of *Sarcophaga peregrina* (Diptera: Sarcophagidae) Larvae at 25 °C Using ATR-FTIR Spectroscopy and Differential Gene Expression Analysis

**DOI:** 10.3390/insects17070678

**Published:** 2026-06-30

**Authors:** Chengxin Ye, Xiangyan Zhang, Yang Bai, Fengqin Yang, Lei Zhao, Yadong Guo, Yanjie Shang

**Affiliations:** 1 Department of Forensic Science, Xiangya School of Basic Medical Sciences, Central South University, Changsha 410013, China; 2 Institute of Forensic Science, Ministry of Public Security, Beijing 100038, China

**Keywords:** *Sarcophaga peregrina*, forensic entomology, minimum postmortem interval, wandering stage, larval age estimation, spectral fingerprinting, chemometric regression, molecular validation

## Abstract

Estimating the post-feeding developmental time of necrophagous larvae remains challenging because body size and behavior provide limited resolution after larvae cease feeding. This study integrated behavioral observation, attenuated total reflectance Fourier transform infrared spectroscopy (ATR-FTIR), chemometric regression modeling, transcriptomic analysis, and reverse transcription quantitative polymerase chain reaction (RT-qPCR) validation to characterize post-feeding development in *Sarcophaga peregrina* (Robineau-Desvoidy) under 25 °C. The post-feeding stage began at 84.83 ± 1.34 h after larviposition and lasted 38.21 ± 2.29 h. ATR-FTIR spectra from 0, 10, 20, 30, and 40 h post-feeding larvae showed temporal changes in the 1800–900 cm^−1^ fingerprint region, mainly involving bands associated with proteins, lipids, polysaccharides, and chitin. Principal component analysis (PCA), partial least squares discriminant analysis (PLS-DA), and variable importance in projection (VIP) analyses identified discriminative spectral regions, while regression models further evaluated time-estimation performance. Among the models, partial least squares regression (PLS-R) showed the most balanced predictive ability, with the coefficient of determination in prediction (R^2^P) = 0.920 and the root mean square error of prediction (RMSEP) = 4.00 h in the prediction set. Transcriptomic analysis identified 4641 differentially expressed genes, and RT-qPCR confirmed temporal expression patterns of selected candidate genes. These molecular changes were mainly associated with cuticle/chitin remodeling, protein metabolism, and energy metabolism. Overall, ATR-FTIR combined with chemometric modeling provides a complementary biochemical approach for estimating post-feeding developmental time in *S. peregrina* under controlled 25 °C conditions, although validation at other temperatures is required before broader application.

## Simple Summary

Estimating how long a person has been dead is an important task in forensic investigations. Insects that develop on remains can provide valuable timing information, but some stages are difficult to estimate accurately. This is especially true after fly larvae stop feeding and leave the food source to search for a place to pupate, because their body size and behavior may no longer change in a simple or reliable way. In this study, we examined this stage in the flesh fly *Sarcophaga peregrina*, an insect species of forensic importance. We used behavioral observations, an infrared light-based method that records chemical fingerprints of the larvae, and gene analysis to follow changes during development. The infrared spectra changed regularly over time and could be used to estimate how long larvae had been in the post-feeding stage, with the best model showing an average prediction error of about 4 h under controlled laboratory conditions. Gene results supported that these chemical changes were related to body remodeling before pupariation. This approach may provide investigators with an additional tool for estimating larval age when post-feeding larvae are found at a death scene.

## 1. Introduction

Estimating the minimum postmortem interval (PMI_min_) is a central task in forensic entomology [1]. Necrophagous flies are among the earliest and most informative colonizers of decomposing remains, and the developmental age of their immature stages provides a biological basis for estimating the time elapsed since colonization [2,3,4]. Current PMI_min_ estimation based on insect evidence relies mainly on species identification, developmental stage determination, larval body size, accumulated thermal units, and standardized interpretation of entomological evidence. However, estimation accuracy depends strongly on developmental data for each species, environmental conditions, and the reliability of age indicators at a given developmental stage. Therefore, reducing uncertainty in developmental age estimation remains a major challenge in forensic entomology.

The post-feeding stage, also referred to as the wandering stage, is a transitional period between active larval feeding and pupariation, during which larvae cease feeding, leave the food source, and disperse in search of suitable pupation sites [5,6]. Compared with feeding larvae, post-feeding larvae no longer show a simple linear increase in body length; instead, body size may decrease because of gut emptying, depletion of stored nutrients, water loss, and pre-pupal contraction [7,8]. Behavioral traits such as locomotion, dispersal, and burial can help define broad developmental intervals, but they are affected by substrate, lighting, temperature, slope, soil conditions, and other environmental contexts [5,9]. Thus, although post-feeding behavior has forensic relevance, it mainly supports broad interval definition rather than precise developmental time estimation.

Several approaches have been explored to improve developmental time estimation beyond conventional morphological observation, including internal tissue examination, gene expression analysis, cuticular hydrocarbons, chromatography and mass spectrometry methods, and spectral approaches based on reflectance [10,11,12,13,14]. These approaches have expanded the methodological basis of forensic entomology and have provided valuable developmental information. However, many of them remain procedurally complex or are influenced by selected targets, sample preservation, extraction procedures, marker selection, and reference data for each species or developmental stage [10,12]. Gene expression approaches, for example, can identify developmentally regulated markers, but they usually require well-preserved samples, RNA extraction, primer design, and species-specific sequence information, which may restrict rapid application in routine forensic settings [15,16,17]. In addition, most available models have been developed for feeding larvae, pupae, or intra-puparial stages, whereas objective biochemical characterization of the post-feeding larval stage remains limited. Therefore, a complementary approach capable of capturing overall biochemical changes, and potentially allowing faster analytical readout after sufficient reference models have been established, would be valuable for post-feeding developmental time estimation.

Attenuated total reflectance Fourier transform infrared spectroscopy (ATR-FTIR) provides molecular fingerprint information based on the vibrational characteristics of chemical functional groups. This fingerprint-based measurement has practical relevance in forensic contexts because spectra can be acquired rapidly with relatively limited sample preparation and without RNA extraction, primer design, or target-specific amplification. The biological fingerprint region, especially 1800–900 cm^−1^, contains information related to lipid esters, protein Amide I/II/III bands, nucleic acids, carbohydrates, polysaccharides, and chitin-associated structures. The general analytical basis of FTIR has been well established for molecular recognition and biological sample characterization [18]. In forensic science, ATR-FTIR has been recognized as a rapid and minimally destructive analytical technique for the characterization of diverse biological and trace evidence [19]. By recording molecular fingerprints related to proteins, lipids, carbohydrates, and nucleic acids, ATR-FTIR can provide chemical information from limited samples with little pretreatment [19]. It has been used for forensic biological traces such as body fluid identification, bloodstain ageing, and species discrimination [20,21,22]. In forensic entomology, infrared spectroscopy has been proposed as a promising tool because insect tissues and cuticular structures contain proteins, lipids, hydrocarbons, and chitin-rich components that change with species, developmental stage, and physiological status [23,24]. These biochemical properties make ATR-FTIR suitable for detecting developmental variation during the post-feeding stage. Combined with chemometric and machine learning models, FTIR spectra can be transformed into discriminative or predictive information for insect identification and developmental age estimation [25,26].

*Sarcophaga peregrina* (Robineau-Desvoidy) is a forensically important flesh fly species with clear relevance to PMI_min_ estimation. It is widely distributed in the Palaearctic, Oriental, and Australasian regions and has been recorded in insect succession studies, human and animal carcasses, and medicolegal autopsy cases [27]. Recent studies have generated genomic, transcriptomic, and temporal gene expression resources for this species, supporting its value in developmental age estimation [28]. Existing studies on *S. peregrina* age estimation have mainly focused on pupal or intra-puparial development [13,17,29,30,31]. However, ATR-FTIR characterization of the earlier post-feeding larval stage remains limited, leaving a methodological gap for a transitional period that is difficult to estimate precisely using body length, morphology, or behavior alone.

In this study, we investigated the post-feeding development of *S. peregrina* under 25 °C by integrating behavioral observation, ATR-FTIR spectroscopy, chemometric regression modeling, transcriptomic analysis, and reverse transcription quantitative polymerase chain reaction (RT-qPCR) validation. Specifically, we aimed to: (i) define the 0–40 h post-feeding developmental window; (ii) characterize temporal spectral changes in the 1800–900 cm^−1^ fingerprint region; (iii) evaluate whether ATR-FTIR regression models can estimate post-feeding developmental time; and (iv) interpret the molecular basis of spectral variation using transcriptomic and RT-qPCR evidence. This integrated framework was designed to provide preliminary species- and temperature-specific evidence for a complementary biochemical approach for post-feeding time estimation in *S. peregrina* under controlled 25 °C conditions.

## 2. Materials and Methods

### 2.1. Insect Rearing and Definition of the Post-Feeding Stage

The necrophagous fly *S. peregrina* was collected from the field in Changsha, Hunan Province, China, and reared for more than five generations under standardized laboratory conditions at 25 °C, 70% relative humidity, and a 12:12 h light/dark cycle. After mating, fresh pig lung was used as the larviposition substrate. The larval rearing procedure was adapted from the method described by Shang et al. [30]. Approximately 1500 larvae produced within 1 h were collected and divided into three groups, with 500 larvae per group. Each group was placed in a small plastic round container (11 cm in diameter and 6.2 cm in height) containing sufficient fresh pig lung. The small container was then placed inside a larger plastic round container (20 cm in diameter and 8.5 cm in height), the bottom of which was covered with sand and the top opening was covered with fine gauze. A representative photograph of the experimental rearing setting has been added as Figure S1. All three rearing containers were maintained in an artificial climate chamber at 25 °C and 70% relative humidity. During the expected transition from the feeding to post-feeding stage and subsequent pupariation, the containers were observed every 4 h. At each observation, larvae that had completely left the pig lung and entered the surrounding sand were counted individually as post-feeding larvae, and immobile individuals with a contracted, pigmented puparium were counted as pupariated. The 50% thresholds for the beginning and end of the post-feeding stage were calculated relative to the initial number of larvae in each container, namely approximately 500 larvae. When more than half of the third instar larvae were observed leaving the food source and entering the surrounding sand, this time point was recorded as the beginning of the post-feeding stage of *S. peregrina*. When more than 50% of the larvae had pupariated, this time point was recorded as the end of the post-feeding stage.

### 2.2. Behavioral Observation and Sample Collection

Larval locomotion was observed from the onset of the post-feeding stage. Behavioral observations were conducted using the three independent rearing groups described above as biological replicate groups. At each 4-h interval, 10 larvae were randomly selected across the three replicate groups, with larvae sampled as evenly as possible from the replicate containers. This number was chosen as a practical balance between obtaining sufficient individual observations for nonparametric comparison and minimizing disturbance to the rearing populations used for subsequent developmental, spectroscopic, and molecular sampling. The selected larvae were placed on white paper covered with a thin layer of sawdust. After the larvae resumed activity, the movement distance within 1 min was recorded. At the end of each observation, the movement tracks left on the sawdust were traced with a marker, and the distance was measured using a thread and ruler. Individual larvae were treated as observational units for locomotion distance analysis. A representative illustration of the locomotion measurement and evaluation procedure has been added as Figure S2. Differences in movement distance among time points were analyzed using the Kruskal–Wallis test, followed by Bonferroni-corrected pairwise comparisons. Statistical significance was set at *p* < 0.05.

For transcriptome sequencing, larvae were collected at 0 h (C1), 12 h (C2), 24 h (C3), and 36 h (C4) after entering the post-feeding stage. At each time point, 10 larvae were collected from each of the three biological replicate groups, resulting in 12 RNA-seq samples and 120 larvae in total.

For RT-qPCR validation and ATR-FTIR analysis, larvae were collected at 0, 10, 20, 30, and 40 h after entering the post-feeding stage. At each time point, 18 larvae were collected from each replicate and equally divided into two groups for candidate gene RT-qPCR validation and ATR-FTIR analysis, respectively. With three independent replicates, 390 larvae were collected for molecular and spectroscopic analyses, including 120 larvae for transcriptome sequencing, 135 for RT-qPCR validation, and 135 for ATR-FTIR analysis. After collection, larvae were rinsed with ultrapure water, dried with filter paper, ground in liquid nitrogen, transferred into 1.5 mL centrifuge tubes, and stored at −80 °C until further analysis.

### 2.3. Attenuated Total Reflectance-Fourier Transform Infrared (ATR-FTIR) Acquisition and Preprocessing

ATR-FTIR spectra were acquired using a Nicolet iN10 FTIR spectrometer (Thermo Fisher Scientific, Waltham, MA, USA) equipped with a germanium ATR accessory. The germanium ATR accessory provides a shallow effective sampling depth, typically below 1 μm in the mid-infrared region, depending on wavenumber, incident angle, and sample refractive index. Before each measurement, the ATR crystal was cleaned with absolute ethanol (Sinopharm Chemical Reagent Co., Ltd., Shanghai, China), and a background spectrum was collected using the empty ATR window. Approximately 2 mg of larval homogenate was loaded onto the germanium ATR crystal for each measurement to completely cover the contact area, and uniform contact between the sample and the crystal surface was ensured using the pressure arm. Spectra were collected over the range of 4000–550 cm^−1^ at a spectral resolution of 4 cm^−1^, with 32 scans per measurement. For each time point, three biological replicate groups were analyzed. From each replicate group, three pooled homogenate samples were measured, and each pooled sample was scanned three times. Therefore, each biological replicate group generated nine raw spectra, each time point generated 27 raw spectra, and a total of 135 raw spectra were obtained from the five post-feeding time points. The three technical scans from the same pooled homogenate sample were first inspected for spectral consistency and artifacts related to contact. Spectra with abnormal baseline drift, excessive noise, or poor contact were reacquired before averaging. The technical scans were then averaged to generate one representative spectrum for each pooled sample, yielding 45 averaged spectra for downstream chemometric and regression analyses.

Raw spectra were exported using OMNIC software (version 9.2; Thermo Fisher Scientific, Waltham, MA, USA). The averaged spectra were then used for preprocessing and modeling. The 1800–900 cm^−1^ fingerprint region was selected for subsequent analysis because this region contains major biochemical information related to lipids, proteins, carbohydrates, polysaccharides, and chitin-associated components. Spectral preprocessing was performed to reduce noise, baseline drift, and scattering effects. Specifically, Savitzky–Golay smoothing with 15 smoothing points was first applied to reduce high-frequency noise. Each spectrum was then normalized using standard normal variate transformation, in which the mean intensity of each individual spectrum was subtracted and the result was divided by its standard deviation. Finally, second-order polynomial baseline correction was performed to minimize baseline drift before multivariate analysis.

### 2.4. Chemometric Analysis and Regression Modeling

Characteristic absorption peaks in the fingerprint region were assigned to major biochemical components according to their molecular vibration features. Principal component analysis (PCA) was first used to explore unsupervised spectral variation among post-feeding time points. Partial least squares discriminant analysis (PLS-DA) was then applied to evaluate supervised discrimination among time points, and wavenumbers with variable importance in projection values greater than 1 were identified as discriminative spectral variables.

Four regression models were established to estimate post-feeding developmental time from the processed spectra from the fingerprint region. The dataset used for regression modeling consisted of 45 averaged spectra, with nine averaged spectra at each of the five post-feeding time points. Data splitting was performed at the pooled sample level to avoid leakage of technical replicates. Within each time point, pooled sample groups 1, 2, 4, 5, 7, and 8 were assigned to the calibration/cross-validation set, whereas groups 3, 6, and 9 were assigned to the holdout test set. Thus, the calibration/cross-validation set contained 30 averaged spectra and the holdout test set contained 15 averaged spectra, with each time point represented in both sets. Partial least squares regression (PLS-R) was used as the primary chemometric regression model. Support vector regression (SVR) with a radial basis function kernel was used as a complementary nonlinear model. Random forest (RF) regression and extreme gradient boosting regression (XGBR) were also performed as comparative machine learning models. Model performance was evaluated using a stratified holdout test set, 50 repeated stratified holdout resampling, and fivefold stratified cross-validation. The coefficient of determination, root mean square error, and mean absolute error were calculated for model evaluation. For the main regression models, 200 y-label permutation tests were performed to assess whether the observed prediction performance exceeded random expectation. Observed versus predicted post-feeding time plots with a 1:1 reference line were used to visualize model performance and potential prediction bias at specific time points. All preprocessing, multivariate analyses, regression modeling, resampling, permutation testing, and visualization were performed using custom Python scripts in Python 3.12.13. The analyses used NumPy 2.4.6, pandas 3.0.1, SciPy 1.17.1, scikit-learn 1.8.0, XGBoost 3.2.0, and joblib 1.5.3. 

### 2.5. Transcriptome Sequencing and RT-qPCR

Total RNA was extracted from *S. peregrina* samples collected at four post-feeding stages (C1–C4) using the Trizol Total Purification Kit I (Beijing ComWin Biotech Co., Ltd., Beijing, China) according to the manufacturer’s instructions. RNA concentration and purity were assessed using a NanoDrop 2000 spectrophotometer (Thermo Fisher Scientific, Wilmington, DE, USA). For each sample, 1 μg of total RNA was used for cDNA library construction with the Illumina^®^ NovaSeq Reagent Kit, and sequencing was performed on the Illumina^®^ NovaSeq 6000 platform (Illumina, San Diego, CA, USA). Paired-end sequencing was performed, and the raw and clean read numbers, Q30 values, total mapping rates, and uniquely mapped rates for each sample are summarized in Table S1.

Raw sequencing reads were filtered using fastp software (version 0.19.5). Clean reads were mapped to the reference genome using hierarchical indexing for spliced alignment of transcripts 2 (HISAT2, version 2.1.0), and gene expression levels were quantified as transcripts per million (TPM) values. Gene-level read counts were used for differential expression analysis. Differentially expressed genes (DEGs) were identified using DESeq2 (version 1.24.0), with the earlier developmental stage used as the reference group. Genes with a false discovery rate (FDR) < 0.05 and |log_2_ fold change| ≥ 1 were considered significantly differentially expressed. Time-related candidate genes were screened by integrating DESeq2-based DEG analysis, weighted gene co-expression network analysis (WGCNA, version 1.63) time-associated modules, Short Time-series Expression Miner (STEM, version 1.3.11) monotonic expression profiles, and K-means time-series clusters. WGCNA was performed using a signed network with soft-thresholding power β = 9, minModuleSize = 30, and mergeCutHeight = 0.25. STEM analysis was performed with 50 model profiles and a significance threshold of *p* < 0.05, and monotonic profiles were selected for further screening. Genes supported by at least two screening strategies and showing monotonic expression patterns with relevant functional annotations were prioritized. Finally, 10 candidate DEGs were selected for RT-qPCR validation.

For RT-qPCR validation, total RNA was extracted from samples collected at 0, 10, 20, 30, and 40 h after entering the post-feeding stage for qPCR validation. cDNA was synthesized using the Evo M-MLV Reverse Transcription PreMix Kit (Accurate Biology, Changsha, China), and RT-qPCR was performed using the SYBR Green Pro Taq HS PreMix Kit with ROX (Accurate Biology, Changsha, China) on an ABI 7500 Real-Time PCR System (Applied Biosystems, Foster City, CA, USA). Primers for the candidate DEGs and reference gene were designed based on the transcriptome data of *S. peregrina* (NCBI BioProject: PRJNA1110974) using Primer Premier 5.0 (Biosoft Premier, Palo Alto, CA, USA) and are listed in Table S2. Arpc2 was used as the reference gene. Relative expression levels were calculated using the 2^−ΔΔCt^ method. Statistical analysis of RT-qPCR data was performed using Origin Pro 8.6.

## 3. Results

### 3.1. Observation of Post-Feeding Behavior in S. peregrina

**Figure 1:**
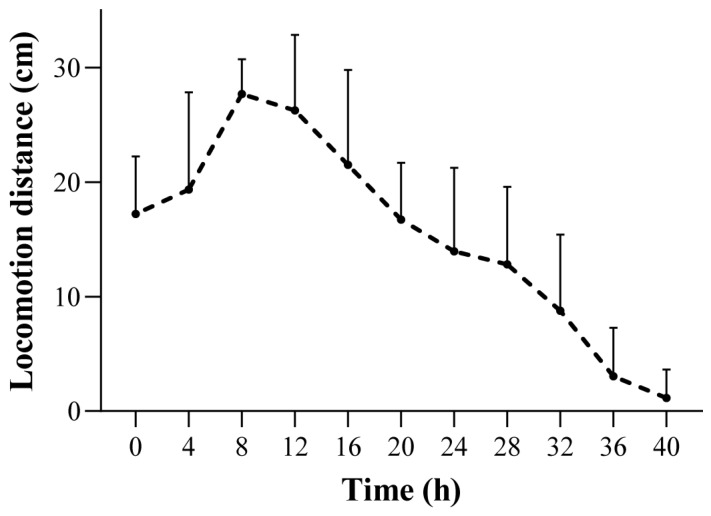
Locomotion distance of *Sarcophaga peregrina* larvae at different developmental times after entering the post-feeding stage. The *x*-axis represents time elapsed after entering the post-feeding stage of *S. peregrina*. The *y*-axis represents the locomotion distance recorded during a 1 min observation period at each time point (10 samples corresponding to each time point). Each point corresponds to the mean movement distance of each group, with error bars indicating the standard deviation of the data for each group.

The post-feeding stage of laboratory-reared *S. peregrina* began at approximately 84.83 ± 1.34 h after larviposition and lasted for approximately 38.21 ± 2.29 h. Observations revealed that the locomotion distance of *S. peregrina* larvae initially increased after entering the post-feeding stage, followed by a gradual decrease until they ceased moving altogether (Figure 1). Under 25 °C conditions, significant differences were observed among all post-feeding observation time points of *S. peregrina* (H_c_ = 70.230, *p* = 4.002 × 10^−11^). After grouping the observations into early, middle, and late post-feeding intervals, locomotion distance also differed significantly among the three intervals. According to the Bonferroni-corrected pairwise comparisons, the locomotion distance during the 0–16 h, >16–28 h, and >28–40 h intervals after entering the post-feeding stage could be well distinguished, with all pairwise adjusted *p*-values being less than 0.05 (Table 1).

### 3.2. ATR-FTIR Spectral Changes During Post-Feeding Development

**Figure 2:**
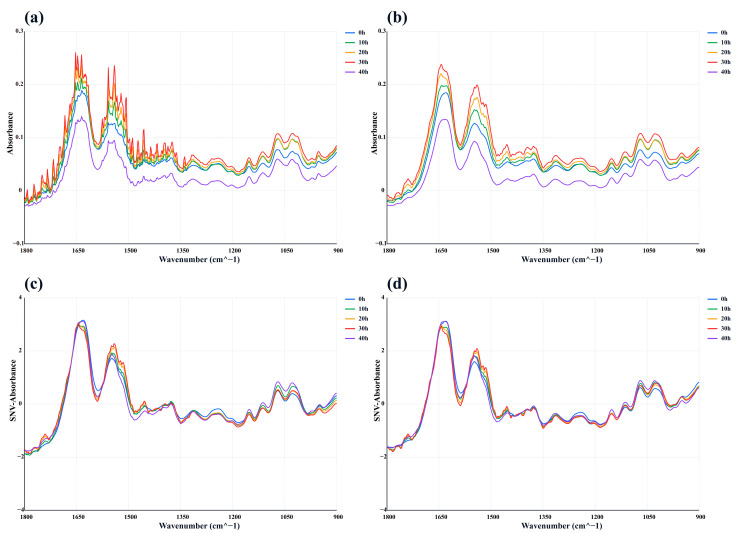
Mean raw spectra (**a**), Savitzky–Golay (SG)-smoothed spectra (**b**), standard normal variate (SNV)-transformed spectra (**c**), and baseline-corrected spectra (**d**) of *S. peregrina* larvae at different post-feeding time points from 0 to 40 h.

Figure 2 shows the average ATR-FTIR spectra of *S. peregrina* larvae at 0, 10, 20, 30, and 40 h after entering the post-feeding stage within the 1800–900 cm^−1^ fingerprint region. The raw spectra displayed several distinct absorption bands (Figure 2a). Savitzky–Golay smoothing effectively reduced high-frequency noise while preserving the major spectral profiles (Figure 2b). After SNV transformation and baseline correction, the comparability of spectra among time points was improved, and relative differences were mainly observed in protein-related regions and carbohydrate/polysaccharide-rich regions (Figure 2c,d). The main absorption peaks were located at approximately 1745, 1648, 1544, 1463, 1403, 1230, 1160, and 1063 cm^−1^. These bands were mainly assigned to C=O stretching vibrations of lipid esters, protein Amide I/II/III bands, C–H and C–OH/O–H vibrations, and C–O–C/C–O vibrations of carbohydrates, polysaccharides, and chitin-related components (Table 2). These results indicate that post-feeding development of *S. peregrina* is accompanied by temporal biochemical changes involving proteins, lipids, and polysaccharide/chitin-related components.

### 3.3. Chemometric Discrimination of Post-Feeding Time Points

**Figure 3:**
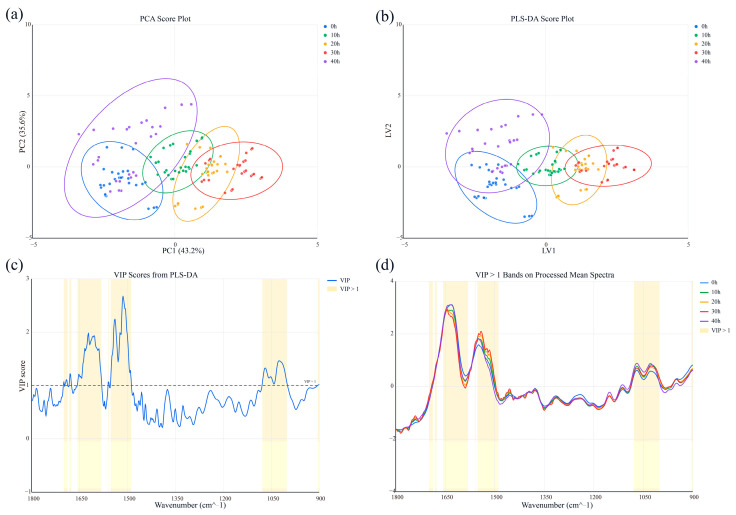
Chemometric analysis of ATR-FTIR spectra from *S. peregrina* larvae at different post-feeding time points. (**a**) Principal component analysis (PCA) score plot of spectra from the 0, 10, 20, 30, and 40 h groups. (**b**) Partial least squares discriminant analysis (PLS-DA) score plot based on latent variables (LV) 1 and 2, showing temporal group separation. (**c**) Variable importance in projection (VIP) scores from the PLS-DA model, with the dashed line indicating the VIP = 1 threshold. (**d**) Processed mean spectra with VIP > 1 regions highlighted.

Principal component analysis (PCA) was first performed to explore the unsupervised distribution of FTIR spectra among the five post-feeding time points. As shown in Figure 3a, the first two principal components explained 78.8% of the total variance, with PC1 and PC2 accounting for 43.2% and 35.6%, respectively. The spectra showed an overall temporal distribution pattern: the 0 h group was mainly located on the negative side of PC1, the 10 h and 20 h groups were distributed in the intermediate region, and the 30 h group shifted toward the positive side of PC1. However, partial overlap was observed among adjacent time points and between the 0 h and 40 h groups, indicating that unsupervised PCA could reveal spectral changes during post-feeding development but was insufficient to completely distinguish all time points.

Supervised discriminant models were then applied to further improve group separation. Compared with PCA, the PLS-DA score plot based on the first two latent variables showed improved clustering according to post-feeding time, but substantial overlap remained among neighboring time points (Figure 3b). The PLS-DA model showed only moderate discriminative performance, with R^2^Y = 0.482, Q^2^ = 0.458, and a cross-validated accuracy of 0.681. A permutation test further indicated that the model performed better than random expectation (*p* for Q^2^ = 0.005). To identify the spectral variables contributing most to time-point discrimination, VIP scores were calculated based on the PLS-DA model. As shown in Figure 3c, wavenumbers with VIP > 1 were mainly distributed in protein-related regions around 1700–1600 cm^−1^ and 1560–1500 cm^−1^, as well as in carbohydrate/polysaccharide-rich regions around 1080–1000 cm^−1^ and near 900 cm^−1^. When these VIP regions were mapped onto the processed mean spectra, their locations largely corresponded to the spectral regions showing visible differences among post-feeding time points (Figure 3d). These discriminative bands were mainly associated with Amide I, Amide II, lipid/carbonyl-related vibrations, and C–O/C–O–C vibrations of carbohydrates, polysaccharides, and chitin-related components. Together, the PCA, PLS-DA, and VIP analyses indicate that post-feeding development of *S. peregrina* is accompanied by systematic spectral changes, particularly in protein- and polysaccharide/chitin-related biochemical components.

### 3.4. FTIR-Based Regression Models for Post-Feeding Time Estimation

**Figure 4:**
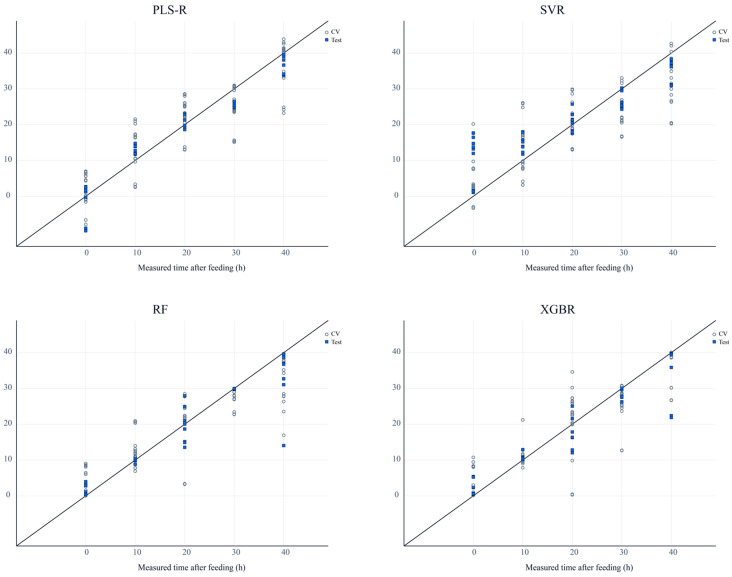
Observed versus predicted post-feeding time of *S. peregrina* larvae based on ATR-FTIR spectra using partial least squares regression (PLS-R), support vector regression (SVR), random forest (RF) regression, and extreme gradient boosting regression (XGBR) models. Open circles represent cross-validation results, blue squares represent holdout test set predictions, and the diagonal line represents the 1:1 reference line.

Regression models were established based on the processed spectra from the fingerprint region to evaluate the ability of FTIR spectra to predict post-feeding developmental time. As shown in Figure 4, the predicted values from all four models showed positive agreement with the measured post-feeding time, although the degree of dispersion around the 1:1 reference line varied among models. Among the four models, PLS-R showed the best overall predictive performance. In cross-validation, PLS-R achieved an R^2^CV of 0.802 and an RMSECV of 6.29 h. In the prediction set, it achieved an R^2^P of 0.920, an RMSEP of 4.00 h, and an MAEP of 3.24 h. The predicted values from PLS-R were closely distributed around the 1:1 reference line, indicating good agreement between measured and predicted values. RF regression and XGBR also showed good predictive performance, with R^2^P values of 0.883 and 0.880, RMSEP values of 4.85 h and 4.90 h, and MAEP values of 2.30 h and 3.02 h, respectively. In contrast, SVR showed relatively weaker performance, with an R^2^CV of 0.664, an RMSECV of 8.19 h, an R^2^P of 0.767, an RMSEP of 6.83 h, and an MAEP of 5.13 h (Table 3). Overall, PLS-R provided the most balanced predictive performance, while RF regression and XGBR produced comparable prediction results. The RMSEP of 4.00 h corresponded to approximately 10% of the 0–40 h post-feeding interval. These findings indicate that FTIR spectral data contain sufficient temporal information for estimating the post-feeding developmental time of *S. peregrina* within the 0–40 h interval.

### 3.5. Transcriptomic and RT-qPCR Evidence Supporting Time-Dependent Biochemical Remodeling

RNA-seq analysis was performed on 12 samples representing four post-feeding developmental stages of *S. peregrina*. Sequencing generated 45.99–61.54 million raw reads and 45.78–61.21 million clean reads per sample, with an average of 51.03 million clean reads per sample. After filtering, the sequencing data showed high quality, with a minimum Q30 ratio of 93.63% and an average Q30 ratio of 94.27%. The alignment rates to the reference genome ranged from 84.31% to 94.37%, with an average mapping rate of 91.21%, and the uniquely mapped rates ranged from 76.02% to 85.48%, indicating that the data were suitable for subsequent transcriptomic analysis (Table S1). PCA and sample correlation clustering further showed overall transcriptional differences among developmental stages and acceptable consistency among biological replicates (Figures S3 and S4).

**Figure 5:**
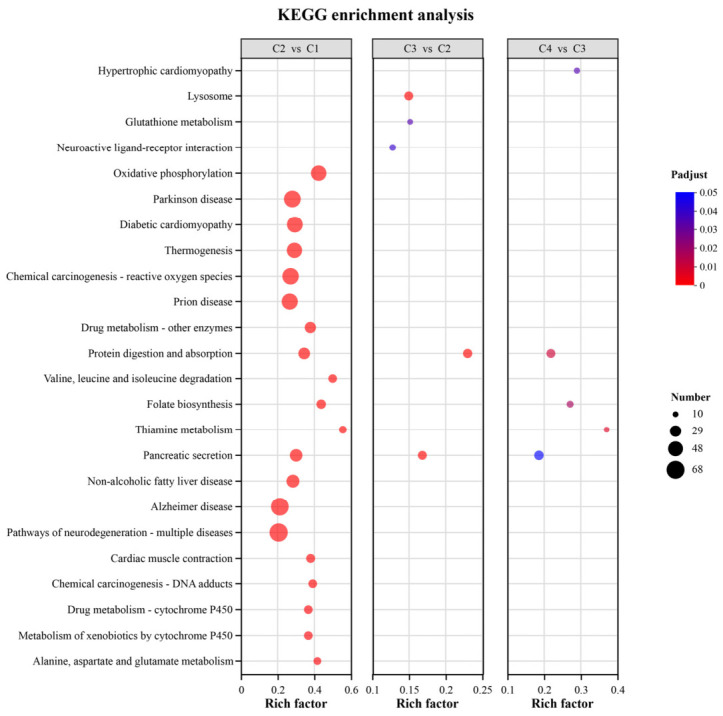
Kyoto Encyclopedia of Genes and Genomes (KEGG) significant enrichment analysis (Top 20) for differentially expressed genes (DEGs) during post-feeding stage.

Differential expression analysis identified 4641 differentially expressed genes (DEGs) during the post-feeding stage. Specifically, 1731 DEGs were identified between C2 and C1, 591 between C3 and C2, and 1328 between C4 and C3 (Figure S5). A total of 75 DEGs were shared among adjacent developmental comparisons, suggesting continuous transcriptional changes during post-feeding development. Hierarchical clustering of DEGs showed stage-related expression patterns across post-feeding samples (Figure S6). Functional enrichment analysis showed that these DEGs were mainly associated with cuticle formation, chitin-related processes, protein metabolism, and energy metabolism. Representative Gene Ontology (GO) terms included “structural constituent of cuticle”, “chitin binding”, “extracellular region”, “alkaline phosphatase activity”, and “serine-type endopeptidase activity” (Figure S7). Kyoto Encyclopedia of Genes and Genomes (KEGG) analysis further showed enrichment of pathways including oxidative phosphorylation, protein digestion and absorption, lysosome, thiamine metabolism, and folate biosynthesis (Figure 5).

**Figure 6:**
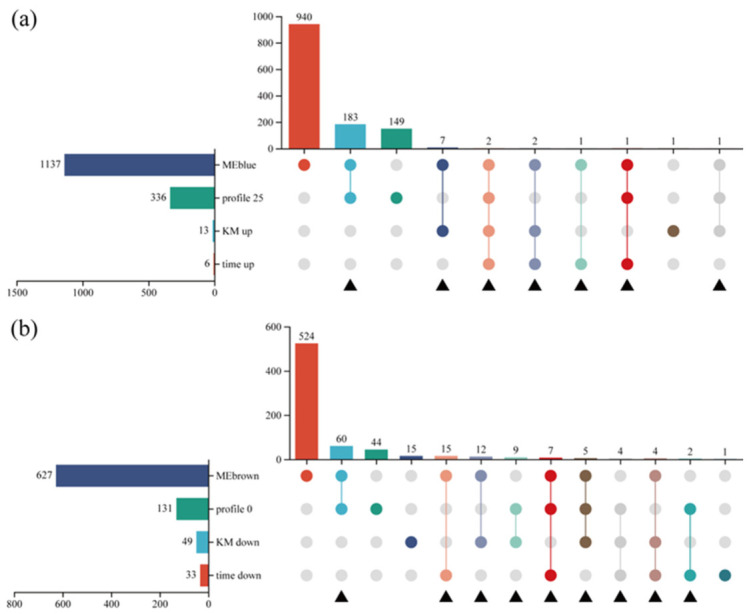
Upset plot of gene sets for differentially expressed genes (DEGs). (**a**,**b**) show intersections among gene sets identified by DEG analysis, weighted gene co-expression network analysis (WGCNA), and time-series analyses. The *y*-axis displays the gene sets involved in the Venn analysis, with the bar graphs showing the number of genes in each gene set. The colored dots and lines on the right side of each column represent the gene sets involved in the Venn analysis. The bar graphs on the *x*-axis correspond to the number of genes included in each intersection below. The triangle symbol (▲) indicates genes that have been confirmed through at least two methods, including WGCNA and temporal trend analysis, to potentially exhibit monotonic upregulation and downregulation trends during the post-feeding stage.

WGCNA identified eight gene co-expression modules, among which the blue module showed the strongest positive correlation with post-feeding time, whereas the brown module showed a negative correlation. Time-series expression analysis further identified genes with monotonic expression patterns during post-feeding development (Figures S8 and S9). By integrating WGCNA and time-series analysis, 1287 potentially monotonically upregulated genes and 702 potentially monotonically downregulated genes were identified. Among them, 197 upregulated genes and 118 downregulated genes were supported by at least two screening methods (Figure 6). Functional annotation showed that the downregulated genes mainly encoded larval cuticle proteins, trypsins, and peritrophins, whereas the upregulated genes mainly encoded heat shock proteins, Toll-like receptors, and coatomer subunits.

**Figure 7:**
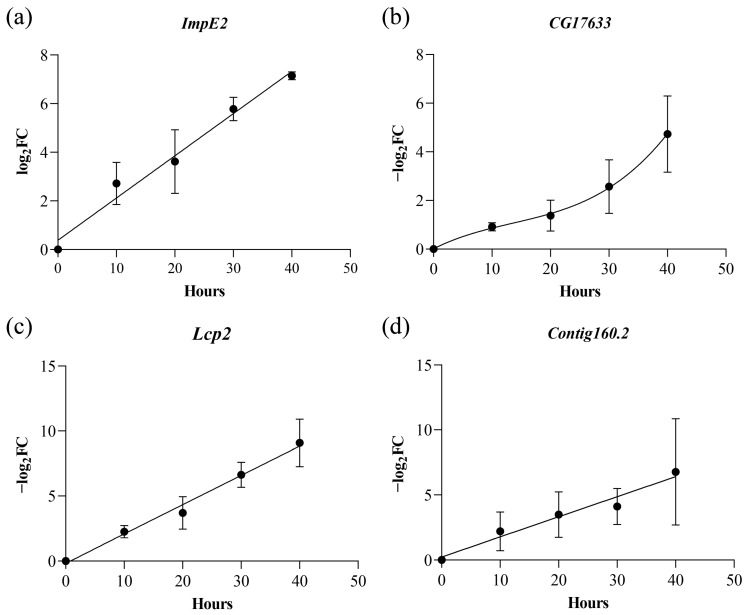
Expression trend changes and fitted equations of target genes during the post-feeding stage. (**a**–**d**) depict the expression trend changes and fitted equations of *ImpE2*, *CG17633*, *Lcp2*, and *Contig160.2*. The *y*-axis represents the standardized log2 fold change (log_2_FC) expression level for differentially expressed genes (DEGs). Each point corresponds to the mean of the standardized expression level log_2_FC, with error bars indicating the standard deviation of the data for each group.

Based on the integrated screening results, functional annotation, and monotonic expression patterns, 10 candidate DEGs were selected for RT-qPCR validation (Table S2). Candidate time-related genes were validated using samples collected at 0, 10, 20, 30, and 40 h after entering the post-feeding stage. Seven genes showed expression trends consistent with the transcriptomic results and exhibited monotonic changes. Among them, *ImpE2*, *Hsp23*, and *Contig80.97* showed increasing trends, whereas *Cht10*, *Lcp2*, *CG17633*, and *Contig160.2* showed decreasing trends. Further analysis showed that *ImpE2*, *Lcp2*, *CG17633*, and *Contig160.2* exhibited statistically significant temporal expression changes (Figure 7). Based on the relative expression levels of these four genes, gene expression-time fitting models were further constructed. All fitted models showed good fit, with R^2^ values > 0.90 and *p* values < 0.05 (Table S3), among which *Lcp2* showed the best fitting performance with post-feeding time.

Overall, the transcriptomic and RT-qPCR results indicate that post-feeding development of *S. peregrina* is accompanied by coordinated molecular remodeling involving proteins, cuticle/chitin-related components, and metabolic pathways. These molecular changes are consistent with the FTIR spectral differences observed in protein- and polysaccharide/chitin-associated regions.

## 4. Discussion

The behavioral observations in this study confirmed that the post-feeding stage of *S. peregrina* at 25 °C represents a short but clearly transitional developmental window, during which larval activity declined from active dispersal to complete immobility. This pattern is consistent with previous studies showing that post-feeding larvae modify their locomotion, dispersal distance, and burial behavior while searching for pupation sites; for example, Robinson et al. reported that indoor surface type affected the dispersal activity and development of post-feeding *Lucilia sericata* (Meigen) larvae [9], and Ferreira et al. showed that post-feeding Sarcophagidae larvae display species-specific mobility and burial behaviors relevant to forensic interpretation [5]. However, these behavioral traits mainly divide development into broad intervals and are sensitive to substrate, environment, and larval physiological state, limiting their use as precise age indicators. This agrees with the broader view that insect evidence should be interpreted using standardized developmental data and objective analytical tools rather than relying solely on visual or behavioral observations [4,32,33]. Previous work has attempted to improve developmental time estimation using statistical growth models and gene expression markers; Tarone and Foran used generalized additive models to quantify uncertainty in *L. sericata* growth estimation, and later showed that developmentally regulated gene expression could improve age estimation precision [34,35]. For *S. peregrina*, recent studies have mainly focused on larval to pupal development, intra-puparial morphology, DEG expression, ATR-FTIR, and cuticular hydrocarbons for pupal or intra-puparial age estimation [13,30]. Compared with these studies, the present work shifts the target to the earlier post-feeding larval stage and demonstrates that FTIR biochemical fingerprints, supported by transcriptomic and RT-qPCR evidence, can provide a more objective basis for estimating post-feeding developmental time than behavioral observation alone.

Previous work using reflectance spectra has shown that insect specimens contain useful spectral information for forensic age and species determination. Voss et al. used reflectance profiles to determine the age and species of blowfly puparia [14]. The FTIR results further indicate that the post-feeding stage is accompanied by systematic biochemical changes in proteins, lipids, and polysaccharide/chitin-related components. Similar spectral regions have been reported in previous insect FTIR studies. Zhang et al. found that the Amide I band in *Phormia regina* (Meigen) puparia decreased during intra-puparial development, suggesting progressive protein-related changes in the puparium [36]. Guo et al. reported characteristic bands associated with lipids, Amide I/II, and carbohydrates in *Chrysomya nigripes* (Aubertin) puparia, and used these spectral features to classify intra-puparial developmental stages [26]. Shang et al. also showed that ATR-FTIR spectra of *S. peregrina* pupae contained age-related information, particularly in protein and hydrocarbon-associated regions [30]. Our findings are consistent with these studies in that the most informative spectral regions were concentrated around Amide I/II/III bands and carbohydrate/polysaccharide-rich regions. However, the biological material differs: previous studies mainly analyzed puparia, pupal tissues, or exuviae, whereas our study used larval homogenates during the post-feeding stage. This difference likely explains why our spectra reflected broader biochemical remodeling, including internal tissue metabolism, cuticle/chitin-related changes, and protein turnover, rather than surface puparial composition alone. Recent FTIR studies on insect larvae and eggs have also shown that immature insect stages contain chemically informative fingerprints related to proteins, lipids, and cuticle-associated components [37,38]. Therefore, our findings extend established FTIR-based biochemical interpretation from pupal age estimation and species identification to the estimation of post-feeding larval developmental time.

The chemometric analyses further show that FTIR spectra contain both discriminative and predictive information for post-feeding time estimation. PCA revealed a time-related distribution but incomplete separation among adjacent time points. Similar results have been reported in previous ATR-FTIR studies: Guo et al. showed partial PCA separation of *C. nigripes* intra-puparial samples but required supervised models for age classification [26], and Hu et al. reported that PCA alone could not clearly separate six Tenebrionidae larval species, whereas supervised models extracted discriminative spectral features more effectively [37]. In the present study, PLS-DA improved the separation among post-feeding time points, suggesting that temporal spectral variation was present but partly masked by overlapping biochemical signals. However, the moderate PLS-DA performance and substantial overlap among neighboring time points indicate that adjacent post-feeding stages could not be clearly classified as discrete spectral groups. This limitation is consistent with the continuous nature of post-feeding development, and supports the use of regression models for direct time estimation rather than relying on categorical discrimination alone.

More importantly, post-feeding time was treated as a continuous outcome, and regression models were established for direct time estimation rather than simple classification into time groups. Previous ATR-FTIR studies in forensic entomology have mainly used discriminant models, such as PLS-DA and RF, for stage or age interval classification [26,36]. By contrast, the present regression framework provides a direct estimate of developmental time within the 0–40 h post-feeding interval. The PLS-R model achieved an RMSEP of 4.00 h in the holdout test set, suggesting finer laboratory resolution than the broad behavioral intervals observed in this study. However, this value should not be interpreted as a universally acceptable error margin for PMI_min_ estimation, because its forensic relevance depends on case context, developmental stage, temperature history, species, diet, and sampling conditions. Existing molecular age-estimation studies in *S. peregrina* have shown that temporal gene and miRNA markers can track postembryonic or pupal development, with polynomial models based on miRNA markers reporting R^2^ values of 0.88–0.99 [17,28]. Together, these studies indicate that both molecular markers and ATR-FTIR spectra can provide quantitative developmental time information, although ATR-FTIR relies on integrated biochemical fingerprints rather than selected temporal markers. Thus, ATR-FTIR may serve as a rapid complementary approach for post-feeding time estimation, pending further validation across developmental stages and environmental conditions. Among the regression models, PLS-R showed the most balanced performance in the present dataset, whereas SVR performed less well. This differs from the study by Hu et al., in which SVM achieved the highest accuracy for stored-product pest species identification [37]. This difference is likely related to the modeling task and data structure: Hu et al. used a larger interspecific classification dataset with stronger spectral differences, whereas the present study involved a regression problem within one species, with a small sample size, continuous time points, adjacent developmental stages, and highly collinear spectral variables. PLS-R is well suited to such high dimensional spectral data because it extracts latent variables that maximize the covariance between spectral predictors and the response variable [39,40]. The use of a held-out prediction set, cross-validation, repeated resampling, and permutation testing further strengthened the reliability of the regression models and reduced concerns regarding overfitting, which is a common issue in spectroscopic and omics-based modeling [41,42].

The transcriptomic and RT-qPCR results provide biological support for the FTIR-based findings by linking the discriminative spectral regions to developmental molecular remodeling. The enrichment of DEGs in cuticle formation, chitin binding, protein metabolism, oxidative phosphorylation, lysosome-related pathways, and protein digestion and absorption is consistent with recent transcriptomic studies showing that the larva-pupa transition involves coordinated regulation of cuticle turnover, hormone signaling, energy metabolism, and tissue remodeling. Ren et al. reported that *S. peregrina* postembryonic development was characterized by stage-specific expression of cuticle structural genes, 20E/JH-related pathways, oxidative phosphorylation, and tyrosine metabolism [28]. Jia et al. also showed that developmental transcriptomes of *Wohlfahrtia magnifica* (Schiner) were enriched in cuticle development, peptidase activity, immune response, and metabolic processes [43]. In *Bombyx mori* (Linnaeus), Yan et al. further demonstrated that stage-specific cuticle formation is accompanied by dynamic expression of cuticular protein genes [44]. These findings are consistent with our FTIR results, in which the most discriminative regions were concentrated in the Amide I/II/III bands and carbohydrate/polysaccharide/chitin-associated bands. At the gene level, *ImpE2* increased during the post-feeding stage, consistent with functional evidence from *Zeugodacus cucurbitae* (Coquillett) showing that *ImpE2* is an ecdysone-related developmental gene whose knockdown alters pupal and adult development [45]. Conversely, *Lcp2* and *Cht10* decreased over time, consistent with cuticle/chitin remodeling before pupariation; recent studies have emphasized that insect chitinases and cuticular proteins regulate chitin matrix formation, degradation, and cuticular organization during molting and metamorphosis [46,47,48]. The *CG17633* gene belongs to the M14 metalloprotease family and is involved in protein hydrolysis processes [49], it is associated with matrix metalloproteinase activity and binding to zinc ions, playing a role in the clearance of larval airway fluid and the development of adult bristle morphology and neuroblast development [50]. Recent molecular age-estimation studies in *S. peregrina* using miRNAs and circRNAs further support the forensic value of temporal molecular markers [17,31]. Thus, unlike studies that use molecular markers as the primary prediction system, our transcriptomic and qPCR results mainly explain why FTIR spectra changed with post-feeding time, supporting the interpretation that the spectral differences reflect endocrine regulation, cuticle/chitin remodeling, and protein turnover rather than technical variation.

From a forensic perspective, this study provides preliminary evidence that FTIR spectroscopy can help characterize and estimate the post-feeding developmental time of *S. peregrina* at 25 °C. Previous studies on *S. peregrina* have established important developmental data under constant and fluctuating temperatures and have evaluated morphology, ATR-FTIR, cuticular hydrocarbons, and molecular markers for age estimation, mainly during pupal or intra-puparial development [13,30]. In comparison, the present study extends FTIR analysis to the post-feeding larval stage and links spectral variation with regression-based time estimation and transcriptomic remodeling. In a PMI_min_ workflow, the estimated post-feeding interval would represent one component of insect developmental age and could be integrated with species identification, earlier larval developmental data, scene temperature history, and conventional entomological interpretation when post-feeding larvae are the oldest specimens recovered. This provides a complementary approach for studying a developmental period in which behavioral and morphological indicators usually provide only broad time ranges. For example, in a potential casework scenario where post-feeding larvae are recovered from the body or nearby substrates such as soil, clothing, bedding, or floor crevices, ATR-FTIR estimation could provide additional information on how long the larvae have remained in the post-feeding stage.

ATR-FTIR may offer practical advantages for forensic implementation once standardized spectral acquisition protocols and validated reference models are available, because spectral measurement can be relatively rapid and does not require RNA extraction, primer design, or target-specific amplification [19,38]. However, the present workflow should not be regarded as a routine casework protocol. Its application would require access to ATR-FTIR instrumentation, trained personnel, and standardized procedures for sample preparation, spectral acquisition, preprocessing, quality control, and model interpretation [51,52]. ATR-FTIR spectra may be influenced by instrument configuration, ATR crystal material, spectral resolution, contact pressure, sample preparation, and preprocessing; therefore, the prediction models developed in this study should be considered specific to this laboratory at this stage. Inter-laboratory application would require harmonized protocols, strategies for calibration transfer, and external validation using independent datasets. In addition, the effects of sample preservation, moisture content, and the interval between collection and spectral acquisition need further evaluation. Thus, the current approach is more suitable for laboratory-based analysis than direct field use at this stage, and field applicability would require simplified sampling procedures, portable or readily accessible instrumentation, and validation using samples that resemble casework samples. The transcriptomic and RT-qPCR analyses were used primarily to support biological interpretation of the FTIR spectral changes, rather than as required components of routine prediction based on FTIR.

The present findings should be interpreted within the experimental conditions of this study. The FTIR models were developed at 25 °C, whereas the developmental rate of necrophagous insects is strongly affected by temperature [4,33]. Accordingly, larvae reared at other temperatures may show different developmental trajectories, spectral patterns, and model performance. Therefore, validation under additional constant and fluctuating temperature conditions would further strengthen its applicability. In addition, the model was developed using five sampled time points within the 0–40 h post-feeding interval, and further testing with independent samples and intermediate time points would help evaluate its predictive stability. As the holdout test set contained only 15 averaged spectra, the predictive results should be interpreted as preliminary and confirmed using larger independent datasets. Future studies should expand the spectral reference dataset across temperatures, populations, diets, and carrion substrates, and compare FTIR workflows using homogenates and intact larvae. Combining FTIR with selected molecular markers may further improve the robustness of post-feeding developmental time estimation for minimum postmortem interval assessment.

## 5. Conclusions

This study integrated behavioral observation, ATR-FTIR spectroscopy, chemometric regression modeling, transcriptomic analysis, and RT-qPCR validation to characterize post-feeding development in *S. peregrina* under controlled 25 °C conditions. ATR-FTIR spectra in the 1800–900 cm^−1^ fingerprint region captured temporal biochemical changes mainly related to proteins, lipids, and polysaccharide/chitin-associated components. Chemometric analyses demonstrated progressive spectral variation among post-feeding time points, and the PLS-R model provided the most balanced performance for estimating post-feeding developmental time. Transcriptomic and RT-qPCR results further supported that these spectral changes reflected coordinated molecular remodeling involving cuticle/chitin-related processes, protein metabolism, and energy metabolism. Overall, these findings indicate that ATR-FTIR spectroscopy, supported by molecular evidence, provides a complementary biochemical approach for estimating the post-feeding developmental time of *S. peregrina* under controlled 25 °C conditions, which may contribute to PMI_min_ assessment by providing a quantitative estimate for the post-feeding segment of larval development when integrated with conventional entomological evidence and validated developmental reference data.

## Figures and Tables

**Table 1:** Locomotion distance of post-feeding larvae within 1 min.

Time (h)	Locomotion Distance (Median, cm)	Locomotion Distance (Inter Quartile Range, cm)	*H* Value	*p*-Adjust
[0, 16] ^a^	24.7	(14.28, 28.83)	60.21	8.45 × 10^−14^
(16, 28] ^b^	13	(8.45, 18.13)		
(28, 40] ^c^	0	(0, 4.70)		

The adjusted significance of Kruskal–Wallis paired test: P_b vs. a_ = 4 × 10^−5^, P_c vs. a_ = 1.28 × 10^−13^, P_c vs. b_ = 2.6 × 10^−4^.

**Table 2:** Tentative assignment of major ATR-FTIR absorption bands in the 1800–900 cm^−1^ fingerprint region of *Sarcophaga peregrina* larvae.

Wavenumber (cm^−1^)	Vibrational Mode	Tentative Biochemical Assignment
1745	C=O stretching	Lipid esters, fatty acids
1648	Amide I, mainly C=O stretching with C–N/N–H contribution	Proteins; protein/chitin-associated amide structures
1544	Amide II, N–H bending coupled with C–N stretching	Proteins; cuticle-associated proteins
1463	C–H bending	lipids, proteins, aliphatic/cuticular hydrocarbon-related components
1403	C=O vibrations of COO− from free fatty acids	Proteins, fatty acids, carboxylate-containing compounds
1230	Amide III and/or PO_2_^−^ asymmetric stretching; C–N/C–O stretching	Proteins, nucleic acids/phosphates, phospholipids
1160	C–O–C/C–O stretching	Polysaccharides, nucleic acids, chitin-related components
1063	C–O stretching	Carbohydrates, polysaccharides, chitin-related components

**Table 3:** Performance of ATR-FTIR regression models for estimating post-feeding developmental time of *Sarcophaga peregrina* larvae.

Model	R2CV	RMSECV (h)	R2P	RMSEP (h)	MAEP (h)
PLS-R	0.8019	6.2948	0.9199	4.0013	3.2356
SVR	0.6645	8.1919	0.7670	6.8263	5.1342
RF	0.7950	6.4028	0.8825	4.8467	2.3033
XGBR	0.7529	7.0305	0.8801	4.8979	3.0151

## Data Availability

The data presented in this study are contained in the manuscript and available on request from the corresponding author. The raw transcriptome data are available in the NCBI BioProject database under accession number PRJNA1110974.

## Supplementary Materials

The following supporting information can be downloaded at: https://www.mdpi.com/article/10.3390/insects17070678/s1, Figure S1. Representative laboratory rearing setup for *Sarcophaga peregrina* larvae, Figure S2. Schematic illustration of the locomotion measurement procedure for post-feeding larvae of *Sarcophaga peregrina*, Figure S3. PCA score plot of transcriptomic samples during the post-feeding stage of *Sarcophaga peregrina*, Figure S4. Sample clustering heatmap based on transcriptomic correlation, Figure S5. Numbers of differentially expressed genes in pairwise comparisons between post-feeding stages, Figure S6. Hierarchical clustering heatmap of differentially expressed genes across post-feeding stages, Figure S7. GO enrichment analysis of DEGs during the post-feeding stage, Figure S8. STEM analysis of temporal gene-expression profiles during post-feeding development of *Sarcophaga peregrina*, Figure S9. Time-series clustering analysis of differentially expressed genes during the post-feeding stage of *Sarcophaga peregrina*, Table S1. Transcriptome quality control data of *Sarcophaga peregrina* post-feeding larvae, Table S2. Primer information of DEGs with Arpc2 as reference gene, Table S3. The fitting equation between the relative expression level of DEGs and the time of post-feeding development.

## References

[1] Bambaradeniya T.B., Magni P.A., Dadour I.R. (2023). A Summary of Concepts, Procedures and Techniques Used by Forensic Entomologists and Proxies. Insects.

[2] Amendt J., Krettek R., Zehner R. (2004). Forensic entomology. Naturwissenschaften.

[3] Villet M.H., Richards C.S., Midgley J.M., Amendt J., Goff M.L., Campobasso C.P., Grassberger M. (2010). Contemporary Precision, Bias and Accuracy of Minimum Post-Mortem Intervals Estimated Using Development of Carrion-Feeding Insects. Current Concepts in Forensic Entomology.

[4] Amendt J., Campobasso C.P., Gaudry E., Reiter C., LeBlanc H.N., Hall M.J.R. (2007). Best practice in forensic entomology—Standards and guidelines. Int. J. Leg. Med..

[5] Ferreira H.R.P., Barbosa T.M., Vasconcelos S.D. (2026). Post-Feeding Larval Mobility and Burial Behaviour of two Forensically Relevant Species, Peckia (Peckia) chrysostoma (Wiedemann) and Peckia (Sarcodexia) lambens (Wiedemann) (Diptera: Sarcophagidae). Neotrop. Entomol..

[6] Komo L., Charabidze D. (2021). Balance between larval and pupal development time in carrion blowflies. J. Insect Physiol..

[7] Li X., Yang Y., Li G., Li H., Wang Q., Wan L. (2014). The effect of dietary fat levels on the size and development of Chrysomya megacephala (Diptera: Calliphoridae). J. Insect Sci..

[8] Matuszewski S., Mądra-Bielewicz A. (2024). Field validation of post-mortem interval estimation based on insect development. Part 1: Accuracy gains from the laboratory rearing of insect evidence. Forensic Sci. Int..

[9] Robinson L.A., Bryson D., Bulling M.T., Sparks N., Wellard K.S. (2018). Post-feeding activity of Lucilia sericata (Diptera: Calliphoridae) on common domestic indoor surfaces and its effect on development. Forensic Sci. Int..

[10] Zhang X., Qu H., Zhou Z., Chen S., Ngando F.J., Yang F., Xiao J., Guo Y., Cai J., Zhang C. (2024). Age Determination of Chrysomya megacephala Pupae through Reflectance and Machine Learning Analysis. Insects.

[11] Stewart-Yates D., Maker G., D’Errico S., Magni P. (2025). Advances and Current Status in the Use of Cuticular Hydrocarbons for Forensic Entomology Applications. Insects.

[12] Shao S., Yang L., Hu G., Li L., Wang Y., Tao L. (2023). Application of omics techniques in forensic entomology research. Acta Trop..

[13] Shang Y., Yang F., Ngando F.J., Zhang X., Feng Y., Ren L., Guo Y. (2023). Development of Forensically Important Sarcophaga peregrina (Diptera: Sarcophagidae) and Intra-Puparial Age Estimation Utilizing Multiple Methods at Constant and Fluctuating Temperatures. Animals.

[14] Voss S.C., Magni P., Dadour I., Nansen C. (2017). Reflectance-based determination of age and species of blowfly puparia. Int. J. Leg. Med..

[15] Lin S.-H., Bellantuono A.J., Lopez K., Wells J.D., DeGennaro M. (2025). Unraveling forensic timelines using molecular markers in Phormia regina maggots. PLoS Genet..

[16] Hartmann K., Herrmann E., Amendt J., Verhoff M.A., Zehner R. (2021). Age-dependent gene expression of Calliphora vicina pupae (Diptera: Calliphoridae) at constant and fluctuating temperatures. Int. J. Leg. Med..

[17] Xia Y., Wu H., Chen S., Wang Y., Sun J., Li Y., Guo Y., Shang Y. (2025). Temporal miRNA Biomarkers for Pupal Age Estimation in Sarcophaga peregrina (Diptera: Sarcophagidae). Insects.

[18] Levin I.W., Bhargava R. (2005). FOURIER TRANSFORM INFRARED VIBRATIONAL SPECTROSCOPIC IMAGING: Integrating Microscopy and Molecular Recognition*. Annu. Rev. Phys. Chem..

[19] Alkhuder K. (2022). Attenuated total reflection-Fourier transform infrared spectroscopy: A universal analytical technique with promising applications in forensic analyses. Int. J. Leg. Med..

[20] Takamura A., Watanabe K., Akutsu T., Ozawa T. (2018). Soft and Robust Identification of Body Fluid Using Fourier Transform Infrared Spectroscopy and Chemometric Strategies for Forensic Analysis. Sci. Rep..

[21] Lin H., Zhang Y., Wang Q., Li B., Huang P., Wang Z. (2017). Estimation of the age of human bloodstains under the simulated indoor and outdoor crime scene conditions by ATR-FTIR spectroscopy. Sci. Rep..

[22] Lin H., Zhang Y., Wang Q., Li B., Fan S., Wang Z. (2018). Species identification of bloodstains by ATR-FTIR spectroscopy: The effects of bloodstain age and the deposition environment. Int. J. Leg. Med..

[23] Barbosa T.M., de Lima L.A.S., dos Santos M.C.D., Vasconcelos S.D., Gama R.A., Lima K.M.G. (2018). A novel use of infra-red spectroscopy (NIRS and ATR-FTIR) coupled with variable selection algorithms for the identification of insect species (Diptera: Sarcophagidae) of medico-legal relevance. Acta Trop..

[24] Jales J.T., Barbosa T.M., de Medeiros J.R., de Lima L.A.S., de Lima K.M.G., Gama R.A. (2021). Infrared spectroscopy and forensic entomology: Can this union work? A literature review. J. Forensic Sci..

[25] Holman A.P., Pickett D.N., West H., Tarone A.M., Kurouski D. (2025). Portable Fourier-transform infrared spectroscopy and machine learning for sex determination in third instar Chrysomya rufifacies larvae. J. Forensic Sci..

[26] Guo Y., Gao Y., Chen N., Tang X., Li L., Hu G., Wang J., Wang Y. (2025). Estimating the Intra-Puparial Period of Chrysomya nigripes Aubertin Using Morphology and Attenuated Total Reflection Fourier Transform Infrared (ATR-FTIR) Spectroscopy. Insects.

[27] Kim J.Y., Lim H.Y., Shin S.E., Cha H.K., Seo J.-H., Kim S.-K., Park S.H., Son G.H. (2018). Comprehensive transcriptome analysis of Sarcophaga peregrina, a forensically important fly species. Sci. Data.

[28] Ren L., Shang Y., Zhang X., Chen S., Zheng Y., Zou Y., Qu Y., Cai J., Zhang C., Guo Y. (2022). Temporal Expression Profiles Reveal Potential Targets during Postembryonic Development of Forensically Important Sarcophaga peregrina (Diptera: Sarcophagidae). Insects.

[29] Li L., Zhang Y., Chen Y., Guo Y., Wang Y., Hu G., Kang C., Jiangfeng W., Wang Y. (2023). Intrapuparial development and age estimation of Sarcophaga peregrina (Diptera: Sarcophagidae) for postmortem interval estimation. J. Asia-Pac. Entomol..

[30] Shang Y., Feng Y., Ren L., Zhang X., Yang F., Zhang C., Guo Y. (2023). Pupal Age Estimation of Sarcophaga peregrina (Diptera: Sarcophagidae) at Different Constant Temperatures Utilizing ATR-FTIR Spectroscopy and Cuticular Hydrocarbons. Insects.

[31] Wu H., Tang H., Han X., Ngando F.J., Shang Y., Guo Y. (2025). Identification of circular RNAs as biomarkers for pupal age estimation and postmortem interval in forensically important Sarcophaga peregrina (Diptera: Sarcophagidae). Int. J. Leg. Med..

[32] Tomberlin J.K., Mohr R., Benbow M.E., Tarone A.M., VanLaerhoven S. (2011). A Roadmap for Bridging Basic and Applied Research in Forensic Entomology. Annu. Rev. Entomol..

[33] Matuszewski S. (2021). Post-Mortem Interval Estimation Based on Insect Evidence: Current Challenges. Insects.

[34] Tarone A.M., Foran D.R. (2008). Generalized Additive Models and Lucilia sericata Growth: Assessing Confidence Intervals and Error Rates in Forensic Entomology. J. Forensic Sci..

[35] Tarone A.M., Foran D.R. (2011). Gene Expression During Blow Fly Development: Improving the Precision of Age Estimates in Forensic Entomology. J. Forensic Sci..

[36] Zhang R., Gao Y., Hu G., Wang Y., Li L., Guo Y., Shao S., Liu S., Wang Y. (2025). Age estimation of Phormia regina pupae based on ATR-FTIR and chemometrics. Spectrochim. Acta Part A Mol. Biomol. Spectrosc..

[37] Hu G., He W., Zhu Z., Li X., Wu J., Zhao L., Guo X., Dai Z., Feng S., Lai Q. (2026). Fourier transform infrared spectroscopy combined with chemometrics: A novel tool for larval species identification of stored-product pests. Spectrochim. Acta Part A Mol. Biomol. Spectrosc..

[38] Zhang R., Wang Y. (2024). Integration of infrared spectroscopy and forensic entomology: A review of three research directions. Appl. Spectrosc. Rev..

[39] Wold S., Sjöström M., Eriksson L. (2001). PLS-regression: A basic tool of chemometrics. Chemom. Intell. Lab. Syst..

[40] Barker M., Rayens W. (2003). Partial least squares for discrimination. J. Chemom..

[41] Devos O., Ruckebusch C., Durand A., Duponchel L., Huvenne J.-P. (2009). Support vector machines (SVM) in near infrared (NIR) spectroscopy: Focus on parameters optimization and model interpretation. Chemom. Intell. Lab. Syst..

[42] Rodríguez-Pérez R., Fernández L., Marco S. (2018). Overoptimism in cross-validation when using partial least squares-discriminant analysis for omics data: A systematic study. Anal. Bioanal. Chem..

[43] Jia Z., Hasi S., Zhan D., Vogl C., Burger P.A. (2024). Transcriptomic profiling of different developmental stages reveals parasitic strategies of Wohlfahrtia magnifica, a myiasis-causing flesh fly. BMC Genom..

[44] Yan Z., Tong X., Xiong G., Yang W., Lu K., Yuan Y., Han M., Hu H., Wei W., Dai F. (2022). A Blueprint of Microstructures and Stage-Specific Transcriptome Dynamics of Cuticle Formation in Bombyx mori. Int. J. Mol. Sci..

[45] Ahmad S., Jamil M., Jaworski C.C., Luo Y. (2023). Comparative transcriptomics of the irradiated melon fly (Zeugodacus cucurbitae) reveal key developmental genes. Front. Physiol..

[46] Dong W., Gao Y.-H., Zhang X.-B., Moussian B., Zhang J.-Z. (2020). Chitinase 10 controls chitin amounts and organization in the wing cuticle of Drosophila. Insect Sci..

[47] Rabadiya D., Behr M. (2024). The biology of insect chitinases and their roles at chitinous cuticles. Insect Biochem. Mol. Biol..

[48] Duan Y., Merzendorfer H., Yang Q. (2026). Molecular Insights into the Biosynthesis of Insect Cuticles. Annu. Rev. Entomol..

[49] Gruntenko N.E., Karpova E.K., Babenko V.N., Vasiliev G.V., Andreenkova O.V., Bobrovskikh M.A., Menshanov P.N., Babenko R.O., Rauschenbach I.Y. (2021). Fitness Analysis and Transcriptome Profiling Following Repeated Mild Heat Stress of Varying Frequency in *Drosophila melanogaster* Females. Biology.

[50] Hosono C., Matsuda R., Adryan B., Samakovlis C. (2015). Transient junction anisotropies orient annular cell polarization in the Drosophila airway tubes. Nat. Cell Biol..

[51] Morais C.L.M., Paraskevaidi M., Cui L., Fullwood N.J., Isabelle M., Lima K.M.G., Martin-Hirsch P.L., Sreedhar H., Trevisan J., Walsh M.J. (2019). Standardization of complex biologically derived spectrochemical datasets. Nat. Protoc..

[52] Baker M.J., Trevisan J., Bassan P., Bhargava R., Butler H.J., Dorling K.M., Fielden P.R., Fogarty S.W., Fullwood N.J., Heys K.A. (2014). Using Fourier transform IR spectroscopy to analyze biological materials. Nat. Protoc..

