# Supplementary material for: Estimating Post-Feeding Developmental Time of Sarcophaga peregrina (Diptera: Sarcophagidae) Larvae at 25 °C Using ATR-FTIR Spectroscopy and Differential Gene Expression Analysis
Source: Insects. 2026 Jun 30;17(7):678. doi: 10.3390/insects17070678 (PMC13410228; doi:10.3390/insects17070678)
Supplement: Supplementary file 1 [file insects-17-00678-s001.zip › insects-4380000-supplementary/Supplementary Figure2.0.pdf]

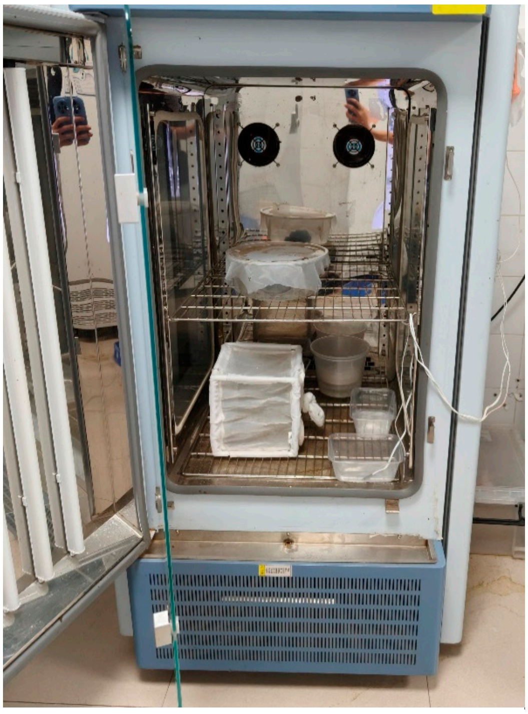

Figure S1. Representative laboratory rearing setup for *Sarcophaga peregrina* larvae.

The system consists of a temperature- and humidity-controlled artificial climate chamber (25 °C, 70% relative humidity), in which larval rearing containers were placed on upper shelf. This image is provided as a representative illustration of the rearing system and does not correspond to a specific experimental sampling moment.

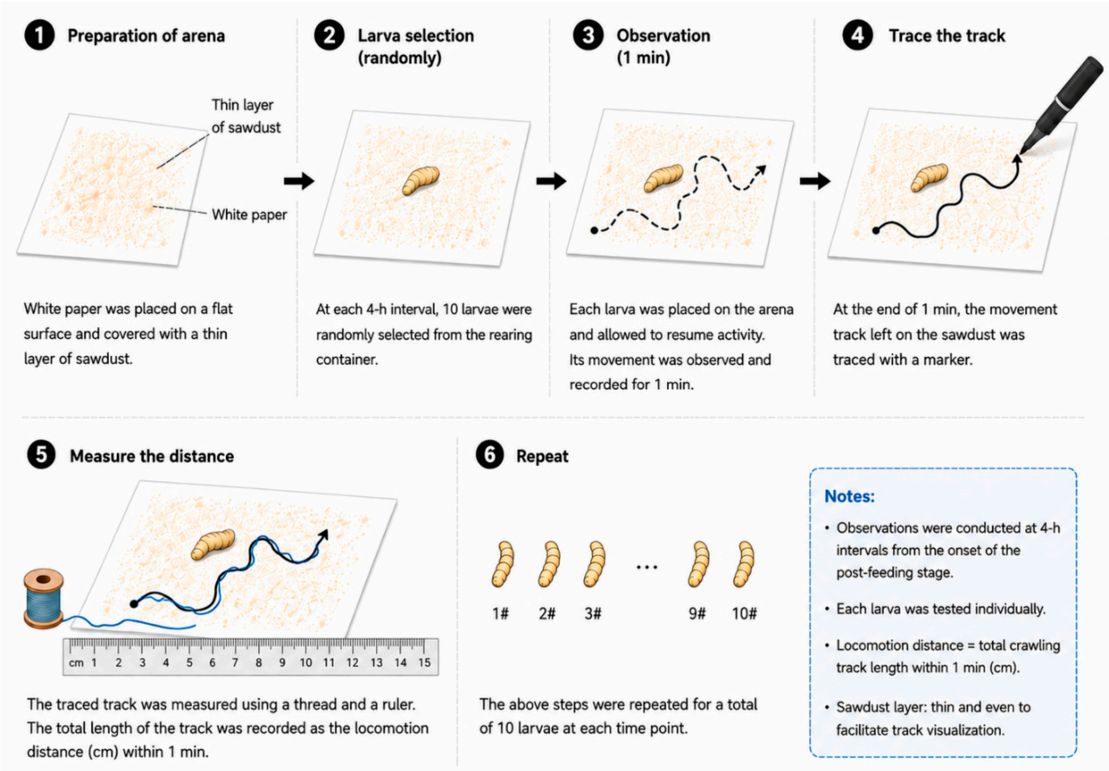

Figure S2. Schematic illustration of the locomotion measurement procedure for post-feeding larvae of *Sarcophaga peregrina*.

Ten larvae were randomly selected at each 4-h interval and assessed individually on white paper covered with a thin layer of sawdust. After each larva resumed activity, the crawling track produced within 1 min was traced with a marker, and the total track length was measured using a thread and ruler.

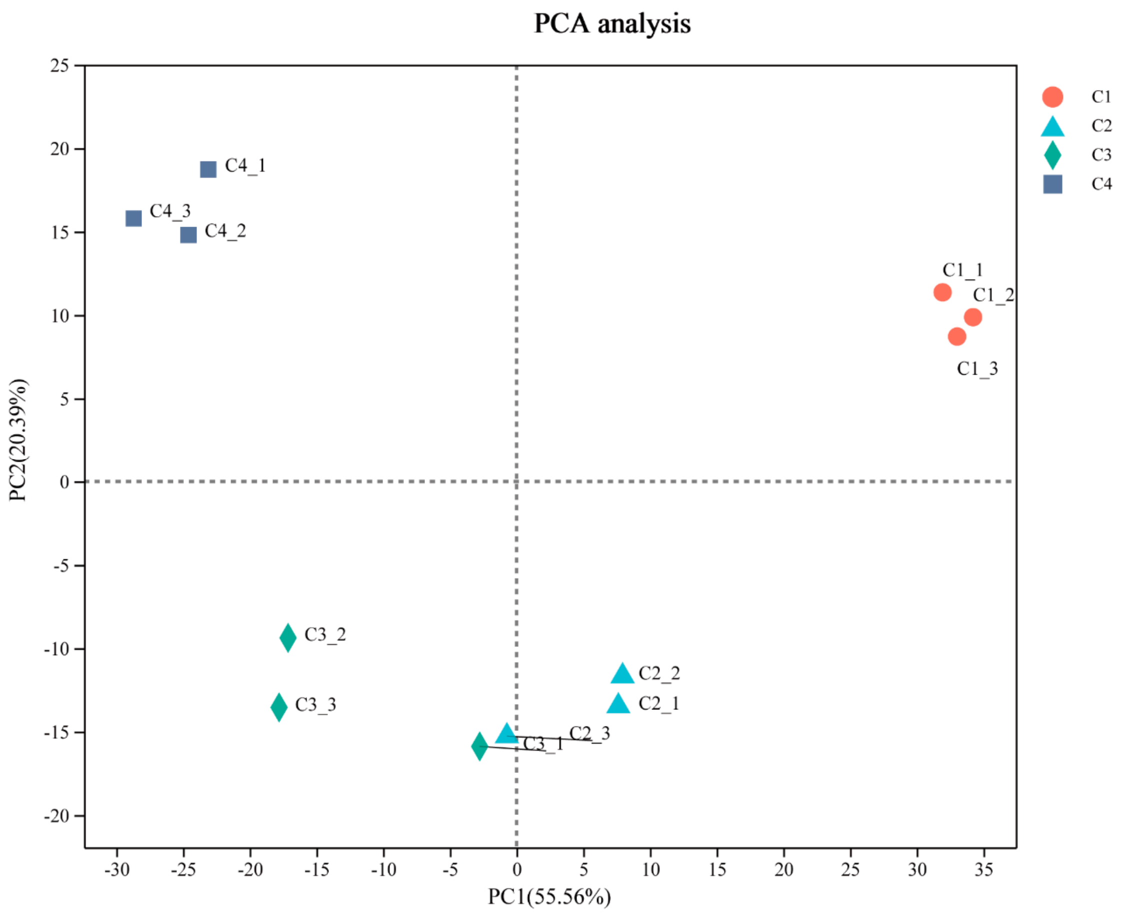

Figure S3. PCA score plot of transcriptomic samples during the post-feeding stage of *Sarcophaga peregrina*.

Different colors represent samples from different post-feeding developmental stages. The distribution of samples indicates the overall transcriptional variation among stages.

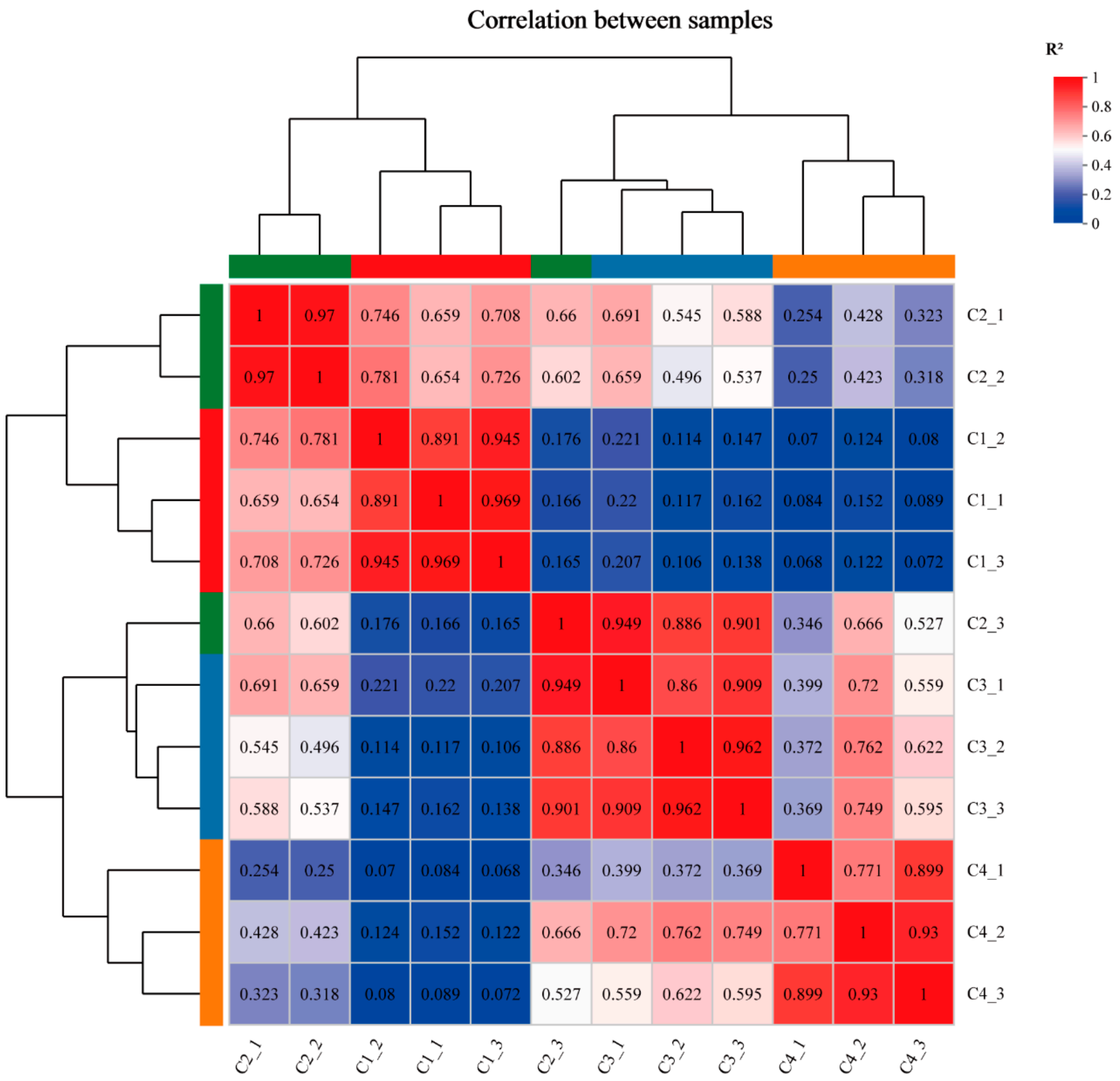

Figure S4. Sample clustering heatmap based on transcriptomic correlation.

The heatmap shows pairwise correlations among RNA-seq samples. Sample names are shown on the right and bottom axes, and the color scale represents the correlation coefficient. Hierarchical clustering of samples is shown on the top and left sides.

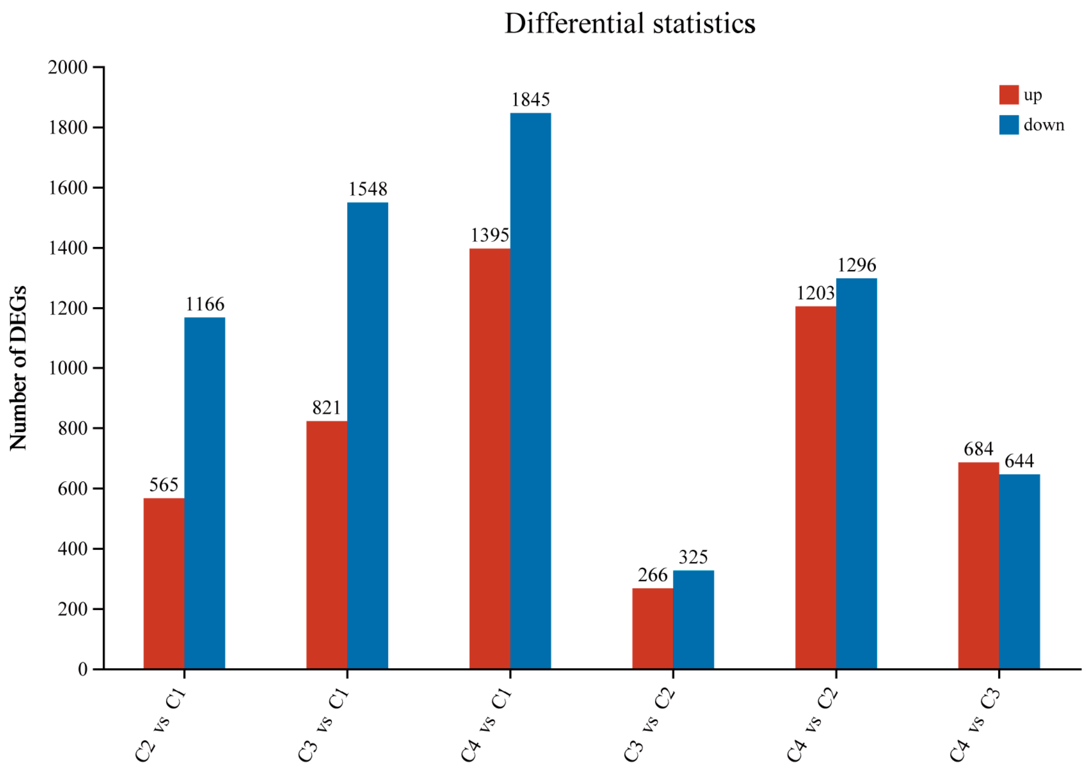

Figure S5. Numbers of differentially expressed genes in pairwise comparisons between post-feeding stages.

The x-axis represents different comparison groups, and the y-axis represents the number of upregulated and downregulated genes.

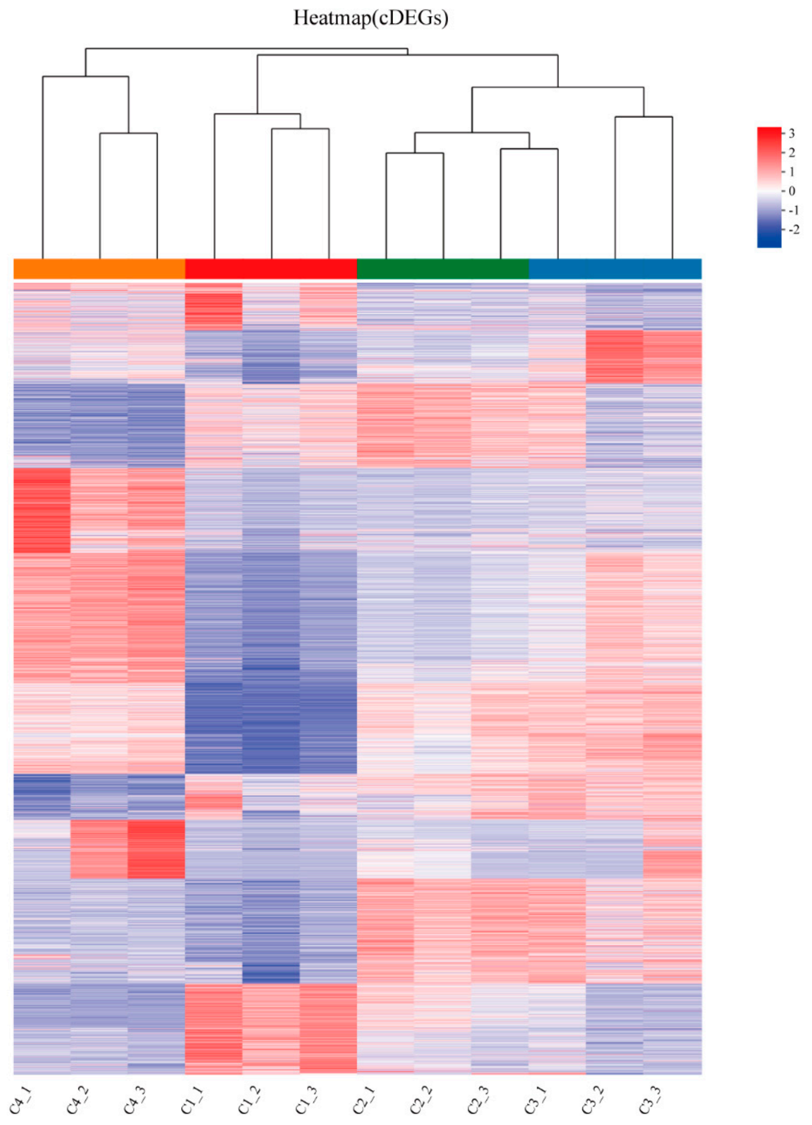

Figure S6. Hierarchical clustering heatmap of differentially expressed genes across post-feeding stages.

Rows represent differentially expressed genes, and columns represent samples. The color scale indicates standardized gene expression levels, and the dendrogram shows clustering based on expression-pattern similarity.

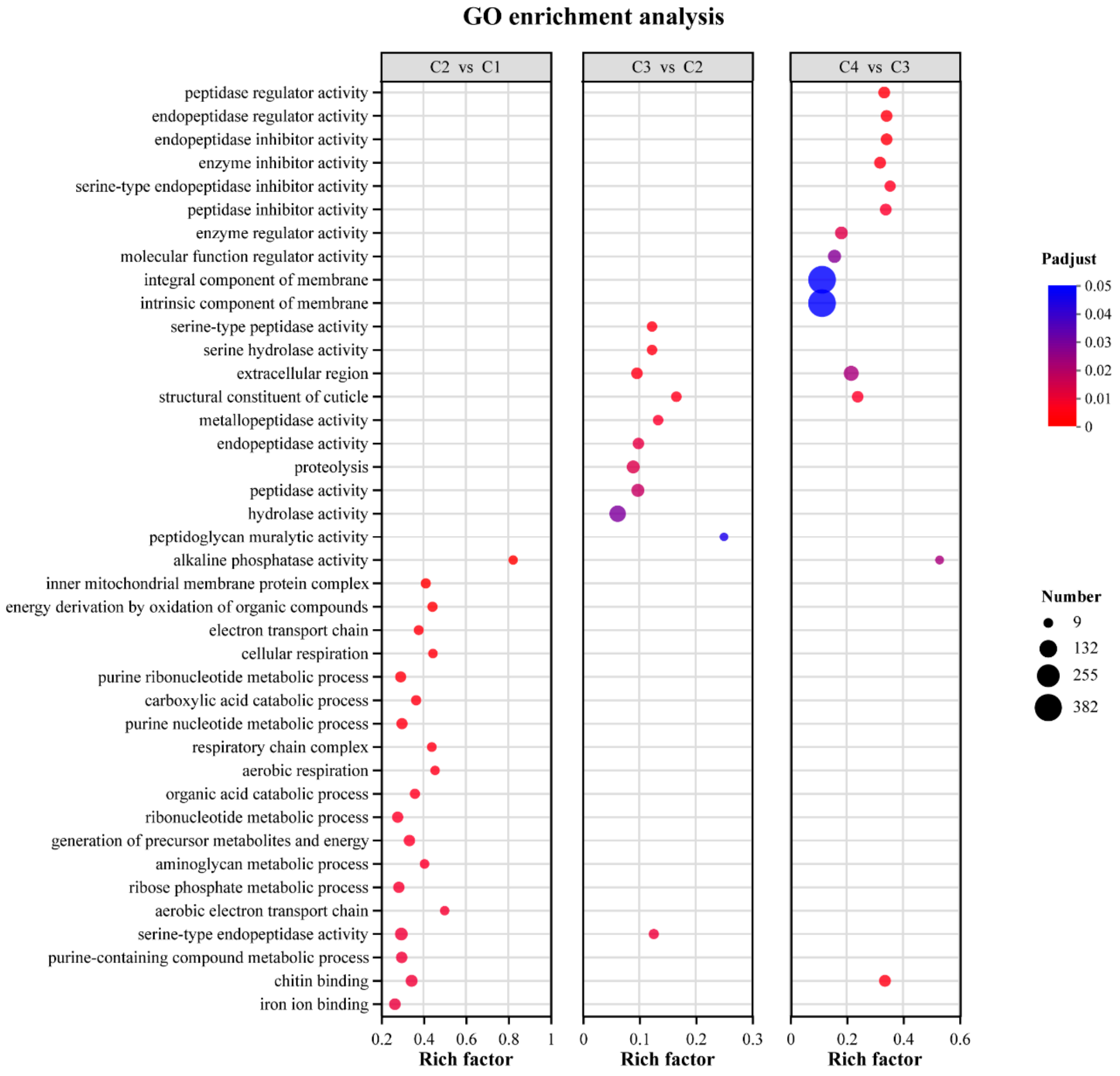

Figure S7. GO enrichment analysis of DEGs during the post-feeding stage.

The top 20 significantly enriched GO terms are shown for each adjacent developmental comparison. The x-axis represents the rich factor, dot size represents the number of enriched genes, and color indicates the adjusted P value.

(a)

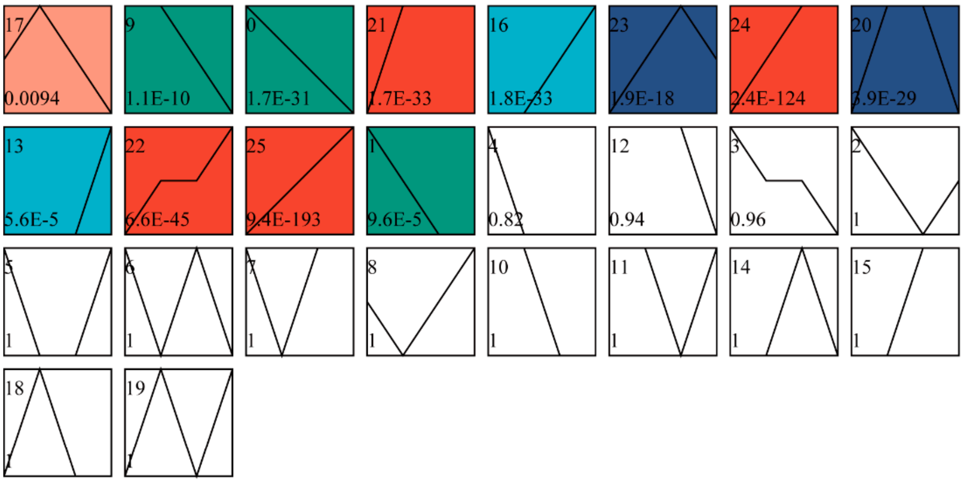

(b)

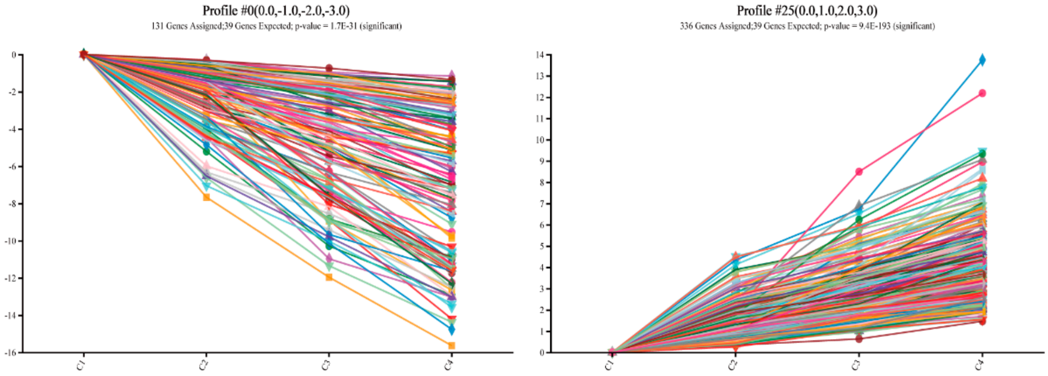

Figure S8. STEM analysis of temporal gene-expression profiles during post-feeding development of *Sarcophaga peregrina*.

(a) Overview of temporal expression profiles, with significant profiles indicated by colored boxes. (b) Detailed expression profiles of genes showing monotonic downregulation and monotonic upregulation across post-feeding stages.

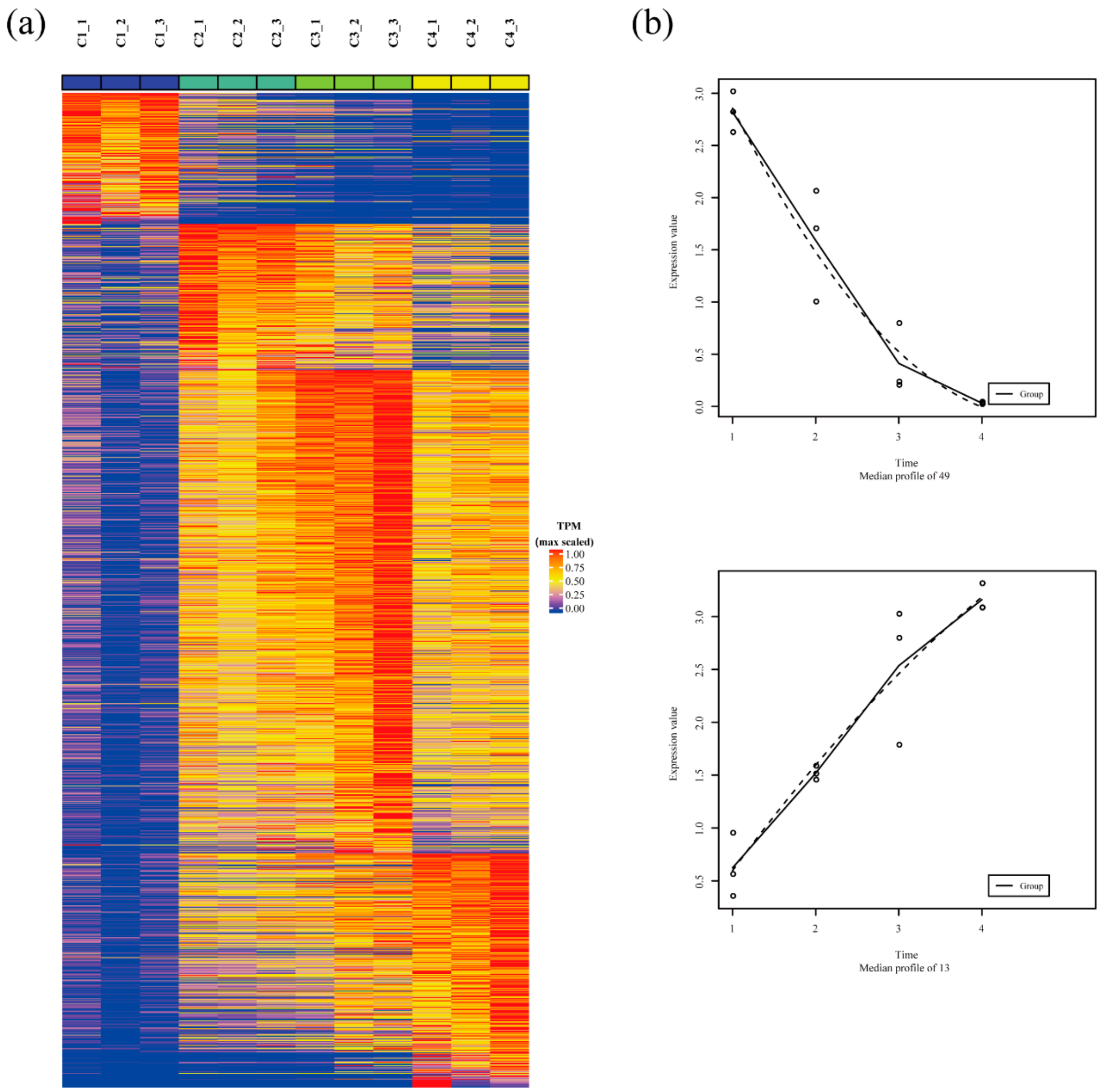

Figure S9. Time-series clustering analysis of differentially expressed genes during the post-feeding stage of *Sarcophaga peregrina*.

(a) Heatmap showing temporal expression patterns of differentially expressed genes across C1–C4. (b) Representative expression patterns of genes showing monotonic downregulation and monotonic upregulation. The x-axis represents developmental time points, and the y-axis represents processed expression values; solid lines connect observed values, and dashed lines indicate fitted trends.
